# Does rating the operation videos with a checklist score improve the effect of E-learning for bariatric surgical training? Study protocol for a randomized controlled trial

**DOI:** 10.1186/s13063-017-1886-7

**Published:** 2017-03-21

**Authors:** Javier Rodrigo De La Garza, Karl-Friedrich Kowalewski, Mirco Friedrich, Mona Wanda Schmidt, Thomas Bruckner, Hannes Götz Kenngott, Lars Fischer, Beat-Peter Müller-Stich, Felix Nickel

**Affiliations:** 1 Department of General, Visceral, and Transplantation Surgery, University of Heidelberg, Im Neuenheimer Feld 110, 69120 Heidelberg, Germany; 2 Institute for Medical Biometry and Informatics, University of Heidelberg, Im Neuenheimer Feld 130.3, 69120 Heidelberg, Germany

**Keywords:** Minimally invasive surgery, Education, Training, Laparoscopy, Human mirror system, Perspective, Serious gaming, First-person view

## Abstract

**Background:**

Laparoscopic training has become an important part of surgical education. Laparoscopic Roux-en-Y gastric bypass (RYGB) is the most common bariatric procedure performed. Surgeons must be well trained prior to operating on a patient. Multimodality training is vital for bariatric surgery. E-learning with videos is a standard approach for training. The present study investigates whether scoring the operation videos with performance checklists improves learning effects and transfer to a simulated operation.

**Methods/design:**

This is a monocentric, two-arm, randomized controlled trial. The trainees are medical students from the University of Heidelberg in their clinical years with no prior laparoscopic experience. After a laparoscopic basic virtual reality (VR) training, 80 students are randomized into one of two arms in a 1:1 ratio to the checklist group (group A) and control group without a checklist (group B). After all students are given an introduction of the training center, VR trainer and laparoscopic instruments, they start with E-learning while watching explanations and videos of RYGB. Only group A will perform ratings with a modified Bariatric Objective Structured Assessment of Technical Skill (BOSATS) scale checklist for all videos watched. Group B watches the same videos without rating. Both groups will then perform an RYGB in the VR trainer as a primary endpoint and small bowel suturing as an additional test in the box trainer for evaluation.

**Discussion:**

This study aims to assess if E-learning and rating bariatric surgical videos with a modified BOSATS checklist will improve the learning curve for medical students in an RYGB VR performance. This study may help in future laparoscopic and bariatric training courses.

**Trial registration:**

German Clinical Trials Register, DRKS00010493. Registered on 20 May 2016.

**Electronic supplementary material:**

The online version of this article (doi:10.1186/s13063-017-1886-7) contains supplementary material, which is available to authorized users.

## Background

Minimally invasive surgery (MIS) plays an important role in a number of surgical disciplines i.e., bariatric surgery. Surgeons require different skills and abilities for MIS compared to open surgery [1]. Over the past two decades, there has been a great patient demand for MIS, requiring laparoscopic training for surgeons [2, 3]. Laparoscopic techniques have created a new paradigm in surgical training. Traditionally, residents and surgeons learned skills hands-on in the operation room (OR), but that approach delays their training in MIS since they are only able to perform few maneuvers [3, 4]. Learning technical and non-technical skills outside the OR is vital for MIS due to additional difficulties that prolong the learning curve. These include pivot and fulcrum effects, lack of haptic feedback, and lack of a three-dimensional view [5]. Currently, there are several laparoscopic training modalities: box trainers, organ models, cadavers, cadaveric organs, live animals, and virtual reality (VR) [6]. With the use of real laparoscopic instruments, box trainers provide a realistic platform for learning [7]. VR has proven to be a safe and effective training modality for MIS, creating a virtual environment for laparoscopic basic skills and operations [7, 8].

The laparoscopic approach to bariatric surgery is considered the “gold standard” for the surgical management of obesity [9]. Laparoscopic Roux-en-Y gastric bypass (RYGB) is the most common bariatric procedure performed [10, 11]. RYGB can be a technically challenging operation for surgeons and trainees. In order to perform the surgery, trainees should first master the basic MIS technique to perform a safe surgery [12]. RYGB has a complication rate that is almost three times higher than suspected during the learning curve [13]. E-learning websites provide videos of surgeries with explanations of the techniques, the relevant anatomy, and perioperative management [14, 15]. The efficacy of E-learning modalities has been studied with positive results for E-learning both alone and in combination with other training modalities [16]. Bariatric Objective Structured Assessment of Technical Skill (BOSATS) is currently the only procedure-specific rating scale specifically developed and validated for use in RYGB. BOSATS was intentionally designed to address multiple approaches to RYGB, increasing its transferability between surgeons and institutions [17]. Checklists, such as BOSATS, have been shown to provide trainees with structured formative feedback and to improve learning curves [18]. Implementation of the BOSATS scale has the potential to provide trainees with objective structured feedback, facilitate deliberate practice, and shorten learning curves in the operating room [17].

We hypothesize that using the BOSATS checklist during E-learning will improve the learning curve and facilitate transfer to practice. The present study aims to explore whether trainees will have an improved learning curve for RYGB on the VR trainer by E-learning and rating videos with a modified BOSATS checklist than just by E-learning without the use of a checklist.

## Methods/design

### Objective

**Fig. 1:**
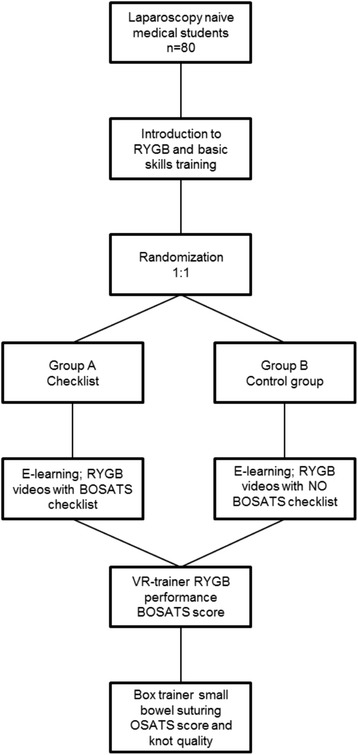
Study protocol flow chart

**Fig. 2:**
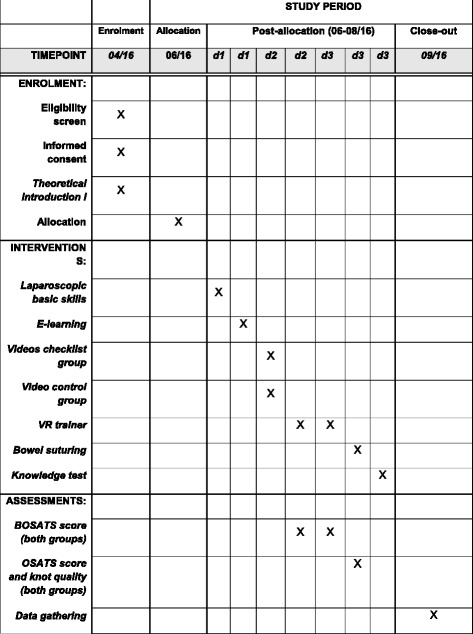
Study process schedule (according to SPIRIT guidelines)

The primary objective of this study is to identify if students in group A, who undergo E-learning and rate surgical videos with a modified BOSATS checklist, will have a better learning curve while performing an RYGB with the VR trainer than students in the control group, who use E-learning without rating the videos. Secondary goals include the transfer of skills to laparoscopic small bowel suturing using an Objective Structured Assessment of Technical Skill (OSATS) scale [19, 20] (Fig. 1). The Standard Protocol Items: Recommendations for Interventional Trials (SPIRIT) schedule is given in Fig. 2.

### Study design

This is a prospective, single-center, two-arm, parallel-group randomized controlled trial.

### Settings and trainees

This study is carried out in the MIS training center of the Department of General, Visceral, and Transplantation Surgery at Heidelberg University Hospital. This study offers voluntary laparoscopic training courses to medical students at Heidelberg University during their clinical years of study (3^rd^ to 6^th^ year).

### Inclusion and exclusion criteria

Inclusion criteria for the study are students enrolled at Heidelberg University Medical School during their clinical years. Exclusion criteria are students who are not in their clinical years or who have already participated in basic laparoscopy training courses for more than 2 hours, who have experience in laparoscopic suturing and knot tying, or who have experience assisting in laparoscopic surgeries for more than 2 hours.

### Training curriculum

This curriculum uses multiple modalities of training to verify and ascertain any advantage in each one. The training groups will participate in a standardized and structured multimodality training curriculum involving E-learning, VR trainer and laparoscopic box trainers. Basic skills are trained with the VR trainer and box trainer in a standardized and structured curriculum (Table 1). For E-learning two different websites are used, www.webop.de and www.websurg.com, and three RYGB videos. During E-learning, group A will rate all three videos with a modified BOSATS checklist (Table 2); the control group (group B) will not be using the checklist. After E-learning, both groups will perform a 4-step RYGB in the VR trainer and will be evaluated with a modified BOSATS scale by an experienced member of the staff (Table 2). As an additional test, using a laparoscopic box trainer, trainees will suture a small bowel incision and will be rated with an OSATS scale (Table 3) to evaluate their performance. Additional to this step, trainees will also be evaluated using a knot quality checklist (Table 4) with a maximum of 5 points. As a last step, all trainees will take a technical knowledge test to evaluate their RYGB post-test knowledge (Table 5, Fig. 1).

**Table 1:** Pre-test: virtual reality trainer laparoscopic basic skills tasks

Basic skills	Excercise
Camera manipulation	The 30° angle camera is used to locate 10 balls and take a photo
Eye-hand coordination	Blue or red color objects have to be touched with the same color instrument tip
Clip applying	Ducts have to be clipped in order to stop water leakage
Clipping and grasping	Ducts have to be grasped and pulled to avoid water leakage
Two-handed maneuvers	Balls have to be grasped from a jelly mass and placed into a jelly bowl with the use of both hands
Cutting	A circular form has to be cut with scissors while retracting it
Electrocautery	Highlighted bands have to be cut with the hook cautery
Peg transfer	Pegs have to be transferred from non-dominant hand to the other hand mid-air and placed on that side of the board and then transferred to the other side the same way

**Table 2:** Bariatric Objective Structured Assessment of Technical Skill (BOSATS) scale

Task/step	1 2	3 4	5
Dissection of the gastro-phrenic ligament (angle of His):			
Pull fundus of stomach down (exposure)	Insufficient retraction; traumatic; insufficient exposure	Satisfactory retraction after some repositioning; suboptimal exposure	Appropriate retraction; optimal exposure
Dissect angle of His close to stomach while keeping tension on fundus	Dissection in incorrect plane; insufficient or too much tension; bleeding	Dissection in correct plane; appropriate tension majority of time; occasional tissue damage, bleeding	Dissection in correct plane; careful handling of tissue; appropriate tension at all times; minimal tissue damage, bleeding
Creation of the gastric pouch:			
Dissect along lesser curvature of stomach approx. 7 cm from the gastro-esophageal junction and keep close to stomach	Incorrect plane; incorrect anatomic location; excessive tissue trauma; bleeding with need of suction	Correct plane developed with some difficulty; moderate tissue damage; bleeding not requiring suction	Correct plane in correct anatomic location developed without difficulty or excessive tissue trauma, bleeding
Create a posterior tunnel	Dissection in incorrect plane; unnecessary force; bleeding requiring suction	Dissection in correct plane; occasional tissue damage; bleeding not requiring suction	Dissection in correct plane; careful handling of tissue, minimal tissue damage, bleeding
Introduce and apply a linear cutting stapler transversely to the stomach	Stapler applied in incorrect orientation; serosal damage to stomach	Stapler applied transversely after multiple repositioning attempts	Stapler applied transversely; no requirement for multiple repositioning attempts; no trauma to stomach wall
Remove all tubes from the stomach before firing the stapler	Not done	Done after delay; with prompting	Done without delay or making sure the tube is not stapled (by movement)
Fire stapler	Uncontrolled fire with excessive pull on the stomach	Controlled fire; some slippage of stomach between jaws	Smooth, controlled fire
Develop a posterior tunnel towards the angle of His	Dissection in incorrect plane; unnecessary force; bleeding requiring suction	Dissection in correct plane; occasional tissue damage; bleeding not requiring suction	Dissection in correct plane; careful handling of tissue, minimal tissue damage, bleeding
Introduce and apply another linear cutting stapler to the stomach	Stapler applied in an incorrect orientation; serosal damage to stomach	Stapler applied correctly; multiple repositioning attempts	Stapler applied correctly; no repositioning required; no trauma to stomach wall
Fire stapler	Uncontrolled fire with excessive pull on the stomach	Controlled fire; some slippage of stomach between jaws	Smooth, controlled fire
Confirm complete transection of stomach	Not confirmed	Confirmed briefly without adequate visualization	Methodical confirmation of complete transection
			Time:
Task/step	1 2	3 4	5
Creation of gastro-jejunal anastomosis:			
Linear stapler technique			
Create a gastrotomy in the gastric pouch	No entry into gastric lumen; poor relation between grasper and energy source; excessively large or small; penetration of posterior bowel wall; bleeding	Entry into gastric lumen; appropriate size; more than 1 attempt required	Entry into gastric lumen; appropriate size; no extra movements required
Location of ligament of Treitz	Not found	Rough movements; poor orientation	Smooth movements; correct orientation
Measure approximately 40–60 cm of jejunum distal to the ligament of Treitz	Length not measured	Measured, however individual measurements not of the same size; poor orientation	Measured methodologically; each measurement of the same size; correct orientation
Create an enterotomy in the Roux limb	No entry into bowel lumen; poor relation between grasper and energy source; excessively large or small; penetration of posterior bowel wall	Appropriate size and entry into bowel lumen; not placed in antimesenteric location	Appropriate size and placement of enterotomy; good relation of grasper and energy source; no extra movements required
Introduce one limb of linear cutting stapler into gastric pouch and the other into Roux limb	Unclear of how to insert the staple device; drives staple jaws blindly into the enterotomies	Inserts the stapler, but lacks appreciation of the ideal angle for insertion	Inserts staple jaws with ease; controlled manner; correct angle
Ensure both limbs are symmetrical before firing the stapler	Does not ensure symmetry, antimesenteric location of stapler before closing of jaws	Limbs either nonsymmetrical or not in antimesenteric border before closure of jaws	Correct symmetry and antimesenteric position before closure of jaws
Fire stapler	Uncontrolled fire with excessive pull on the bowel and widening of enterotomies	Controlled fire; some slippage of bowel from jaws	Smooth, controlled fire; no widening of enterotomies
			Time:
Task/step	1 2	3 4	5
Creation of jejuno-jejunal anastomosis:			
Linear stapler technique			
Create enterotomies in biliopancreatic and Roux limbs	Poor relation between grasper and energy source; excessively large or small; penetration of posterior bowel wall	Appropriate size enterotomy; not placed in antimesenteric location	Appropriate sized and placed enterotomies; no extra movements. Good relation of grasper and energy source
Insert the limbs of linear cutting stapler into the enterotomies in Roux and biliopancreatic limbs	Unclear of how to insert the staple device. Drives staple jaws blindly into biliopancreatic and Roux limbs	Inserts the stapler with hesitation and lacks appreciation of the ideal angle for insertion	Inserts staple jaws with ease; controlled manner; correct angle
Ensure both limbs are symmetrical and stapler in antimesenteric border	Does not ensure limb symmetry and antimesenteric position before enclose of jaws	Limbs either non-symmetrical or not on antimesenteric border before closure of jaws	Correctly ensures symmetry and antimesenteric position before closure of the jaws
Fire stapler	Uncontrolled fire with excessive pull on the bowel and widening of enterotomies	Controlled fire; some slippage of bowel from jaws	Smooth, controlled fire; no widening of enterotomies
			Time:
Help needed during performance	Asks a lot of questions and needed assistance	Few questions and almost no assistance	Few questions but no assistance

**Table 3:** Procedural checklist and Objective Structured Assessment of Technical Skill (OSATS) scale for laparoscopic suturing and knot tying

Procedure assessment and OSATS			Yes/no
Needle position 1	1	Held at one half to two thirds distance from the tip	
	2	Angle 90° ± 20°	
	3	Uses tissue or other instrument for stability	
	4	Attempts at positioning (≤3)	
Needle driving 1 (entry to incision)	5	Entry at 60° to 90° to tissue plane	
	6	Driving with one movement	
	7	Driving needle with wrist suppination	
	8	Single point of entry through tissue	
	9	Removes needle along its curve	
	10	Pull suture through to establish short free end	
	11	Suture placed accurately, on target	
Needle position 2	12	Held at one half to two thirds distance from the tip	
	13	Angle 90° ± 20°	
	14	Uses tissue or other instrument for stability	
	15	Attempts at positioning (≤3)	
Needle driving 2 (entry in incision)	16	Driving with one movement	
	17	Removes needle along its curve	
Techniques of knots	18	Correct C-loop	
	19	Smoothly executed throw, no fumbles	
	20	Knot laid flat without air knots	
	21	Short free end maintained	
	22	Correct inverse C-loop	
	23	Smoothly executed throw, no fumbles	
	24	Knot laid flat without air knots	
	25	Correct third C-loop	
	26	Smoothly executed throw, no fumbles	
	27	Knot laid flat without air knots	
Pulling the suture	28	Needle on needle holder in view at all times	
	29	Uses the pully concept	
	30	Knot squared	
	31	Appropriate tissue reapproximation without strangulation	
	32	Good use of both hands to facilitate knot tying	
General	33	Kept needle in view at all times when grasping	
	34	Non-dominant hand helps dominant hand in suturing

**Table 4:** Knot quality checklist

Knot quality assessment	Available points
No visible gaps between stacked throws	1
Knot tight at base	1
Only edges are opposed (no extra tissue in knot)	1
Knot holds under tension	2
Maximum	5

**Table 5:** Multiple choice knowledge test

1. Which ligament should be dissected as a first step of a laparoscopic Roux-en-Y gastric bypass (RYGB)?	
A) Gastro-colic	C) Gastro-phrenic
B) Spleno-renal	D) Gastro-splenic
2. For the gastric pouch, dissection should begin at the lesser curvature of the stomach _____ cm from the gastro-esophageal junction.	
A) 7 cm	C) 4 cm
B) 10 cm	D) 12 cm
3. For the gastric pouch, a posterior tunnel has to be dissected towards the _________.	
A) Incisura angularis	C) Angle of His
B) Pylorus	D) Spleen
4. For the Roux limb creation, which ligament should be found?	
A) Round ligament	C) Hepato-duodenal
B) Treitz	D) Gastro-colic
5. For the Roux limb creation, what gastrointestinal segment needs to be measured?	
A) Duodenum	C) Jejunum
B) Ileum	D) Colon
6. What is the approximate length of the Roux limb?	
A) 25–35 cm	C) 100 cm
B) 70–90 cm	D) 40–60 cm
7. For the gastro-jejunal anastomosis, what is the location for the jejunum’s enterotomy?	
A) Anterior location	C) Mesenteric location
B) Antimesenteric location	D) Posterior location
8. Which instrument is mainly used for the creation of enterotomies?	
A) Dissector/Maryland	C) Scissors
B) Harmonic scalpel	D) Grasper
9. For the creation of the biliopancreatic limb, does the surgeon measure the alimentary limb?	
A) Yes	B) No
10. How many staple fires are usually required for a jejuno-jejunal anastomosis?	
A) 1 staple	C) 2 staples
B) 3 staples	D) 4 staples

### Introduction to the training modalities in the training center

The trainees receive a standardized introduction and instructions to use the VR trainer, box trainer, and instruments by trained staff. All students can familiarize themselves with the training center and training devices before starting the tests and exercises.

### Basic skills training

All trainees will attend the MIS training center of the Department of General, Visceral, and Transplantation Surgery at Heidelberg University Hospital and perform 10 hours of standardized basic skills training. This includes instrument coordination tasks as well as laparoscopic suturing and knot tying exercises with box trainers. At the end the trainees will perform basic skills tasks with the VR trainer for one hour as a pre-test (Table 1).

### Pre-test

The pre-test for both groups includes the laparoscopic basic skills training tasks in the VR trainer. Groups A and B will perform eight basic skills tasks before starting with E-learning. The objective for these exercises is to learn about the VR trainer management and functions to train for their RYGB performances (Table 1).

### Randomization

Trainees are randomly allocated to either the checklist group (group A) or control group (group B) with the sealed envelopes technique. The randomization of subjects is performed in a 1:1 ratio by block randomization with a variable block length using a computer-generated randomization list. Trainees are allocated to groups without stratification by gender or previous operative experience. The employee responsible for the randomization and group assignment is otherwise not involved with the training, tests, and data from the present study. As student recruitment to the study will be completed before randomization, any influence of randomization results or subsequent task assignments is considered minimal. We aim to compare both groups following data acquisition.

### Introduction to laparoscopic Roux-en-Y gastric bypass by E-learning

All trainees work with E-learning modalities for three hours as an introduction to RYGB after randomization. This is done in a standardized fashion by using the same room at the Department of Surgery at Heidelberg University Hospital with identical surrounding conditions in order to rule out any difference between trainees. The trainees are given an explanatory introduction by trained staff in a standardized way to begin the RYGB modalities on www.webop.de and www.websurg.com. During this introduction, trainees are asked to study and understand the anatomy, illustrations, and videos of the procedural techniques. Following this general overview, the trainees will watch three anonymized RYGB videos to get a clearer view of the surgical techniques. Group A will rate the correct performance of the operative technique with the BOSATS checklist, while group B will not use a checklist (Table 2).

### Post-test

The post-test includes the RYGB on the VR trainer and a modified BOSATS evaluation. Groups A and B will perform the VR trainer post-test at the end of the training curriculum. Both groups will perform RYGB on the VR trainer three times and will be evaluated with the modified BOSATS by an experienced staff member who is blinded to the training status of trainees (Table 2).

### Transfer of training test

The additional test includes suturing a small bowel incision with the laparoscopic technique. After the post-test, groups A and B will suture a 3-cm incision on cadaveric porcine small bowel in a laparoscopic box and will be evaluated by the blinded staff with an OSATS score for suturing and knot tying (Table 3) and a knot quality checklist (Table 4).

### Knowledge test

As a last step, all trainees will take a multiple choice (MC) technical knowledge test to evaluate their knowledge on the RYGB technique after the training curriculum (Table 5).

### Primary endpoint

The primary endpoint is the performance of a 4-step RYGB on the VR trainer based on the modified BOSATS score evaluated by a blinded expert rater [17]. RYGB steps on the VR trainer include (1) dissection of the gastro-phrenic ligament and creation of the gastric pouch, (2) location of Treitz ligament and measurement, (3) creation of gastro-jejunal anastomosis, and (4) creation of the jejuno-jejunal anastomosis.

### Secondary endpoints

The secondary endpoints include the time spent on the VR trainer to perform RYGB; time will be taken at all three times the students perform the procedure. VR trainer subscores and single parameters for each trainee will be evaluated. Also, trainees’ laparoscopic small bowel suturing performance will be included and evaluated with an OSATS scale. Additional endpoints include subgroup analyses of gender differences, gaming experience, and questionnaire evaluations of training after the course. Previous studies state that since surgery has been traditionally a male field, male students acquire surgical skills faster and have superior visuospatial skills than female students [21–24].

### Statistical analysis

For both groups, the distribution of continuous data will be presented using mean, standard deviation (SD), minimum, maximum, and median, and for categorical variables, absolute and relative frequencies will be used. The primary endpoint, which is the modified BOSATS score, will be compared between both groups using a *t* test with a significance level of 0.05. Comparisons regarding secondary endpoints will be performed by the chi-square test for categorical data and the *t* test for continuous variables. Resulting *p* values from secondary analyses will be interpreted descriptively.

### Sample size determination

Sample size determination was calculated for the BOSATS score. Previous published data from a study by Zevin et al. was used. The data was modified according to the BOSATS with a maximum score of 115 points. Group 1 had a mean score of 95.8 points with an SD of 9.9, while group 2 had a mean of 82.9 points with an SD of 15.0. Calculation was done for a significance level of *α* = 0.05 and a power of 1 − *β* = 0.8. An additional 10% was added to each group to compensate for the adjustment of the data. With these data differences can be detected with a minimum of 24 trainees in each group. To account for possible drop-outs the planned group size is 40 trainees per group.

## Discussion

This study evaluates if students who rate videos with a checklist during E-learning will have a better learning curve while performing an RYGB in the VR trainer than those who do E-learning without the ratings and checklist. Rating videos seems like an extra training for students; therefore, expectations are that trainees who perform the video ratings will have a better performance than those who just use E-learning and no rating. The continuous data recording of the VR trainer and the tests will help us understand if there is a difference in learning curves between both training groups [25]. The assessments of the study trainees will help us to understand the possible factors of influence for successful surgical education. It is important to ascertain which module will have a better outcome to be implemented into further laparoscopic and bariatric surgery training.

### Limitations of the study

There are some limitations to the study; subjects are limited to be medical students in their clinical years. Participants’ lack of surgical knowledge and bariatric surgery experience may influence their performance during the study. On the other hand, the inclusion of laparoscopy-naïve medical students allows for better differentiation of intervention effects, as the study group is very homogenous concerning surgical experience. In addition, the students have a total of 11 hours of laparoscopy training using the box trainer and the VR trainer before performing the virtual RYGB after extensive E-Learning for this procedure. Due to the fact that the trainees are laparoscopic novice medical students, the results cannot be transferred directly to more experienced surgeons. However, the results will provide a better perspective for designing optimal bariatric surgery training.

## Trial status

Recruitment started in April 2016 and the collection of data was finished in August 2016. Data analyses are currently running.

## Additional file

Additional file 1: SPIRIT checklist. (DOCX 63 kb)

## Abbreviations

BOSATS: Bariatric Objective Structured Assessment of Technical Skill
MC: Multiple choice test
MIS: Minimally invasive surgery
OSATS: Objective Structured Assessment of Technical Skill
RYGB: Laparoscopic Roux-en-Y gastric bypass
SD: Standard deviation
VR: Virtual reality

## References

[1] Hendrie JD, Nickel F, Bruckner T, Kowalewski K-F, Garrow CR, Mantel M (2016). Sequential learning of psychomotor and visuospatial skills for laparoscopic suturing and knot tying — study protocol for a randomized controlled trial “The shoebox study”. Trials.

[2] Hamad GG, Curet M (2010). Minimally invasive surgery. Am J Surg.

[3] Yiannakopoulou E, Nikiteas N, Perrea D, Tsigris C (2015). Virtual reality simulators and training in laparoscopic surgery. Int J Surg Lond Engl.

[4] Friedman RL, Pace BW (1996). Resident education in laparoscopic cholecystectomy. Surg Endosc.

[5] Seymour NE, Gallagher AG, Roman SA, O’Brien MK, Bansal VK, Andersen DK (2002). Virtual reality training improves operating room performance: results of a randomized, double-blinded study. Ann Surg.

[6] Nickel F, Bintintan VV, Gehrig T, Kenngott HG, Fischer L, Gutt CN (2013). Virtual reality does not meet expectations in a pilot study on multimodal laparoscopic surgery training. World J Surg.

[7] Nickel F, Jede F, Minassian A, Gondan M, Hendrie JD, Gehrig T (2014). One or two trainees per workplace in a structured multimodality training curriculum for laparoscopic surgery? Study protocol for a randomized controlled trial — DRKS00004675. Trials.

[8] Stefanidis D, Heniford BT (2009). The formula for a successful laparoscopic skills curriculum. Arch Surg Chic Ill 1960.

[9] Nguyen NT, Goldman C, Rosenquist CJ, Arango A, Cole CJ, Lee SJ (2001). Laparoscopic versus open gastric bypass: a randomized study of outcomes, quality of life, and costs. Ann Surg.

[10] Livingston EH (2010). The incidence of bariatric surgery has plateaued in the U.S. Am J Surg.

[11] Seki Y, Kasama K (2010). Current status of laparoscopic bariatric surgery. Surg Technol Int.

[12] Sánchez-Santos R, Estévez S, Tomé C, González S, Brox A, Nicolás R (2012). Training programs influence in the learning curve of laparoscopic gastric bypass for morbid obesity: a systematic review. Obes Surg.

[13] Schauer P, Ikramuddin S, Hamad G, Gourash W (2003). The learning curve for laparoscopic Roux-en-Y gastric bypass is 100 cases. Surg Endosc.

[14] Pape-Koehler C, Chmelik C, Aslund AM, Heiss MM (2010). An interactive and multimedia-based manual of surgical procedures: Webop—an approach to improve surgical education. Zentralblatt Für Chir.

[15] Mutter D, Vix M, Dallemagne B, Perretta S, Leroy J, Marescaux J (2011). WeBSurg: an innovative educational Web site in minimally invasive surgery--principles and results. Surg Innov.

[16] Pape-Koehler C, Immenroth M, Sauerland S, Lefering R, Lindlohr C, Toaspern J (2013). Multimedia-based training on Internet platforms improves surgical performance: a randomized controlled trial. Surg Endosc.

[17] Zevin B, Bonrath EM, Aggarwal R, Dedy NJ, Ahmed N, Grantcharov TP (2013). Development, feasibility, validity, and reliability of a scale for objective assessment of operative performance in laparoscopic gastric bypass surgery. J Am Coll Surg.

[18] Regehr G, MacRae H, Reznick RK, Szalay D (1998). Comparing the psychometric properties of checklists and global rating scales for assessing performance on an OSCE-format examination. Acad Med J Assoc Am Med Coll.

[19] Nickel F, Hendrie JD, Stock C, Salama M, Preukschas AA, Senft JD (2016). Direct observation versus endoscopic video recording-based rating with the objective structured assessment of technical skills for training of laparoscopic cholecystectomy. Eur Surg Res Eur Chir Forsch Rech Chir Eur.

[20] Nickel F, Brzoska JA, Gondan M, Rangnick HM, Chu J, Kenngott HG (2015). Virtual reality training versus blended learning of laparoscopic cholecystectomy: a randomized controlled trial with laparoscopic novices. Medicine (Baltimore).

[21] Donnon T, DesCôteaux J-G, Violato C (2005). Impact of cognitive imaging and sex differences on the development of laparoscopic suturing skills. Can J Surg J Can Chir.

[22] Thorson CM, Kelly JP, Forse RA, Turaga KK (2011). Can we continue to ignore gender differences in performance on simulation trainers?. J Laparoendosc Adv Surg Tech A.

[23] Ali A, Subhi Y, Ringsted C, Konge L (2015). Gender differences in the acquisition of surgical skills: a systematic review. Surg Endosc.

[24] Nickel F, Hendrie JD, Kowalewski K-F, Bruckner T, Garrow CR, Mantel M (2016). Sequential learning of psychomotor and visuospatial skills for laparoscopic suturing and knot tying—a randomized controlled trial “The Shoebox Study” DRKS00008668. Langenbecks Arch Surg Dtsch Ges Chir.

[25] Nickel F, Hendrie JD, Bruckner T, Kowalewski KF, Kenngott HG, Müller-Stich BP (2016). Successful learning of surgical liver anatomy in a computer-based teaching module. Int J Comput Assist Radiol Surg.

[26] Chan A-W, Tetzlaff JM, Gøtzsche PC, Altman DG, Mann H, Berlin JA (2013). SPIRIT 2013 explanation and elaboration: guidance for protocols of clinical trials. BMJ.

[27] Schulz KF, Altman DG, Moher D, CONSORT Group (2010). CONSORT 2010 Statement: updated guidelines for reporting parallel group randomised trials. BMC Med.

